# A series of patients with unusual lung cancers with unusual presentations

**DOI:** 10.7196/AJTCCM.2019.v25i2.230

**Published:** 2019-07-31

**Authors:** D M Maphanga

**Affiliations:** Division of Pulmonology, Department of Medicine, University of the Witwatersrand and Charlotte Maxeke Johannesburg Academic Hospital, Johannesburg, South Africa

**Keywords:** lung, cancer

## Abstract

Lung malignancies have become increasingly prevalent. Occasionally, an unusual tumour is diagnosed, or a common tumour type presents unusually. This case report reviews 3 cases of thoracic neoplasm, including two cases of uncommon cancers (primary lung adenoid cystic carcinoma and thoracic desmoplastic small round cell high-grade sarcoma) and an atypical presentation of malignant mesothelioma.

## Background

Pulmonary malignancies continue to increase, affecting an estimated 1.8 million people in 2012, contributing to ~1.6 million deaths globally.^[1]^ The incidence of mesotheliomas is also rising due to occupational exposures, with peaks expected between 2015 - 2025 in the United States.^[2]^ In a South African cohort, as much as 23% of mesothelioma cases were linked to environmental exposures, particularly in Northern Cape Province.^[3]^ With such a significant burden of disease, there is a need to be vigilant of lung malignancies in clinical practice, particularly where the presentation may be atypical.

## Case 1

**Fig. 1:**
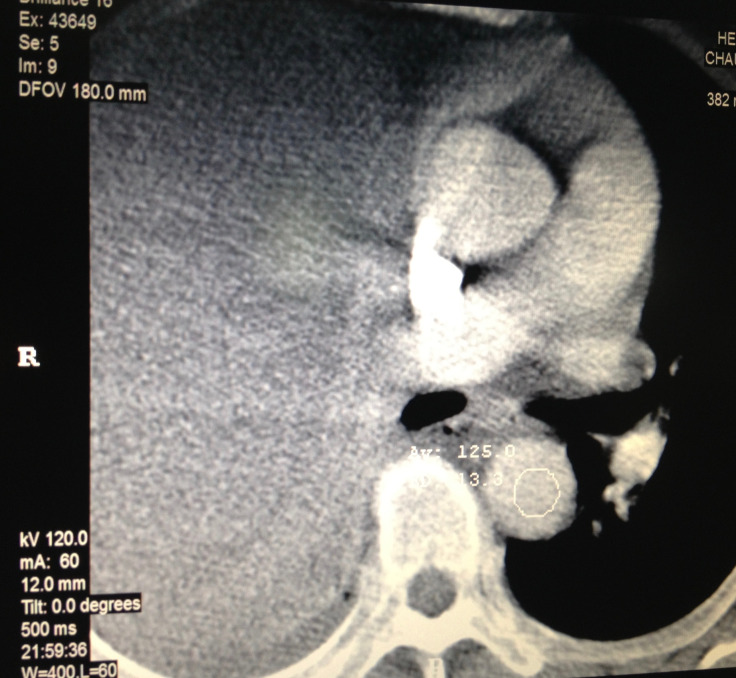
An axial computed tomography scan showing a homogeneously enhancing mass in the right hemithorax with an associated pleural effusion.

Mrs FC is a 56-year-old woman, who presented with a 2-month history of right pleuritic chest pain and haemoptysis. Her chest radiograph and computed tomography (CT) scan revealed an inhomogeneous mass occupying nearly the whole right hemithorax, with an associated small pleural effusion (Fig. 1). Multiple ultrasound guided biopsies were taken which were in keeping with a diagnosis of a malignant mesothelioma (MM).

## Case 2

**Fig. 2:**
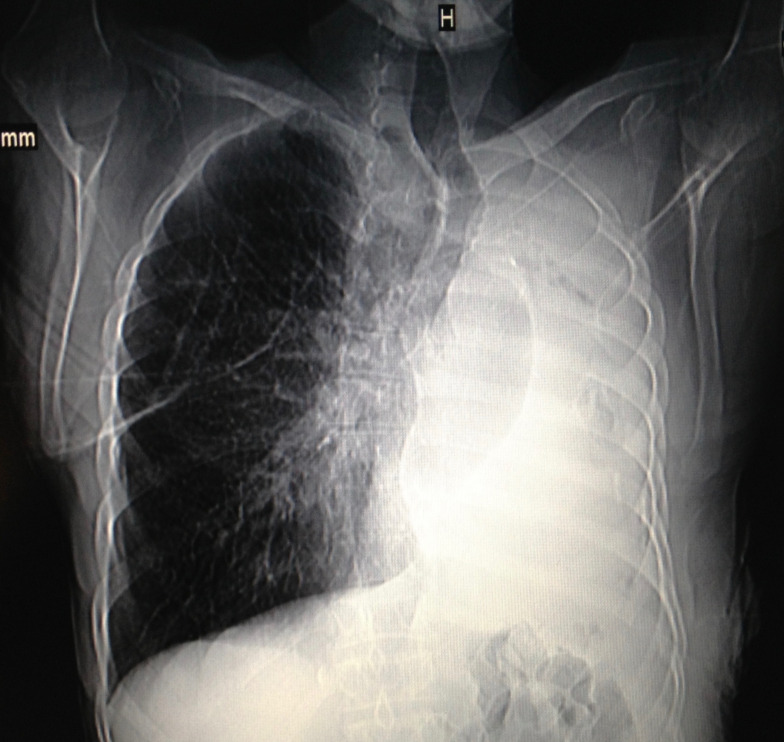
Plain posteroanterior (PA) film of Mr MG showing white-out of the left hemithorax with volume loss (tracheal deviation to the left/rib crowding) and compensatory hyperinflation of the right lung.

**Fig. 3:**
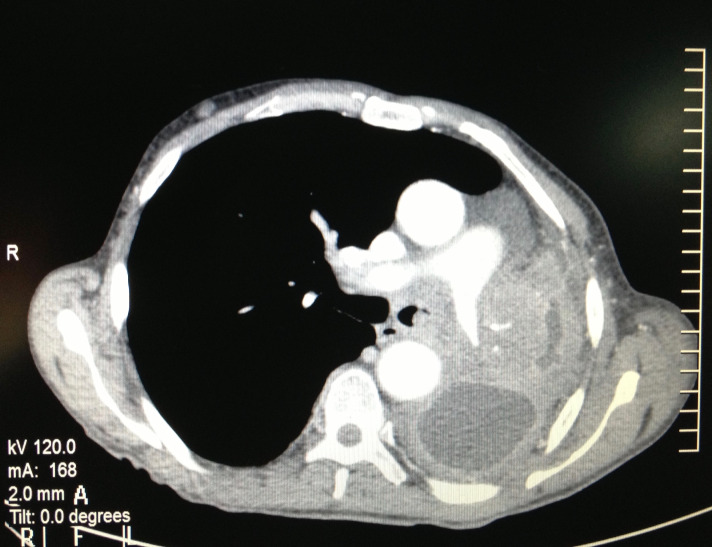
Axial computed tomography images of the chest, showing a left endobronchial mass lesion with total left lung collapse. There is mediastinal deviation to the left. In addition, complex collections are seen in the left hemithorax.

**Fig. 4:**
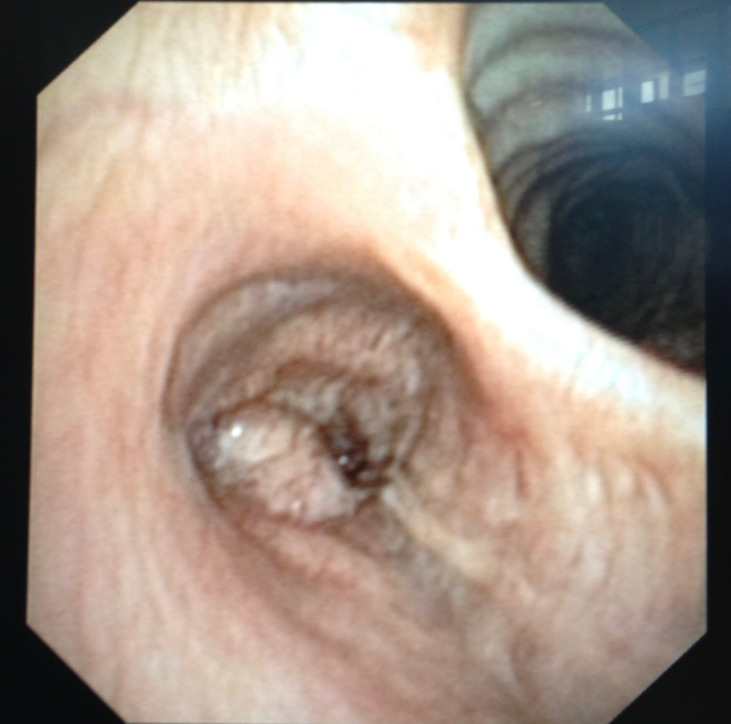
Bronchoscopy images showing an endobronchial mass lesion in the left main bronchus, which occludes the right upper-lobe bronchus.

Mr MG is a 65-year-old man who presented with left-sided chest pain. He was a non-smoker, and also reported profound weight loss. His chest radiograph showed a white-out of the left hemithorax with mediastinal shift. A CT scan revealed the presence of a left main bronchus mass with associated left lung atelactasis and complex collections extending to the chest wall consistent with a left empyema neccesitans. A pigtail catheter was placed to drain the fluid. The purulent fluid aspirated did not yield any organisms on culture. A bronchoscopy was performed which revealed a left upperlobe bronchus that was partially occluded by the tumour. The biopsy confirmed an adenoid cystic carcinoma (ACC) (Figs 2, 3 and 4).

## Case 3

**Fig. 5:**
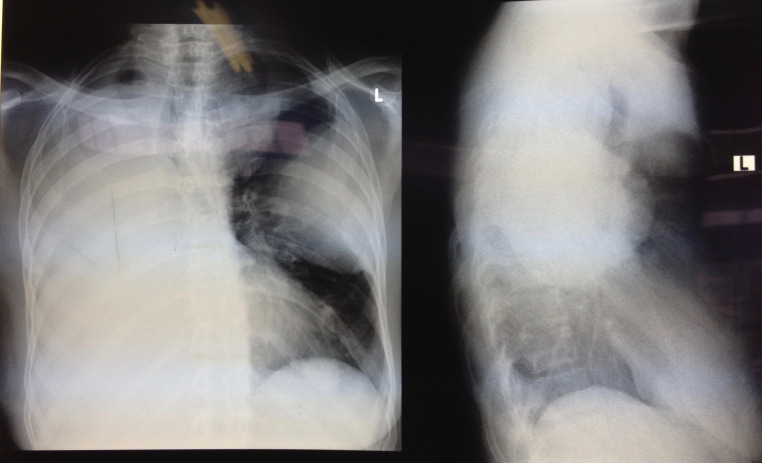
Posteroanterior and lateral plain films of Mrs AM showing nearcomplete white-out of the right hemithorax with tracheal deviation to the right and a well circumscribed rounded radiopaque mass lesion on the left.

**Fig. 6:**
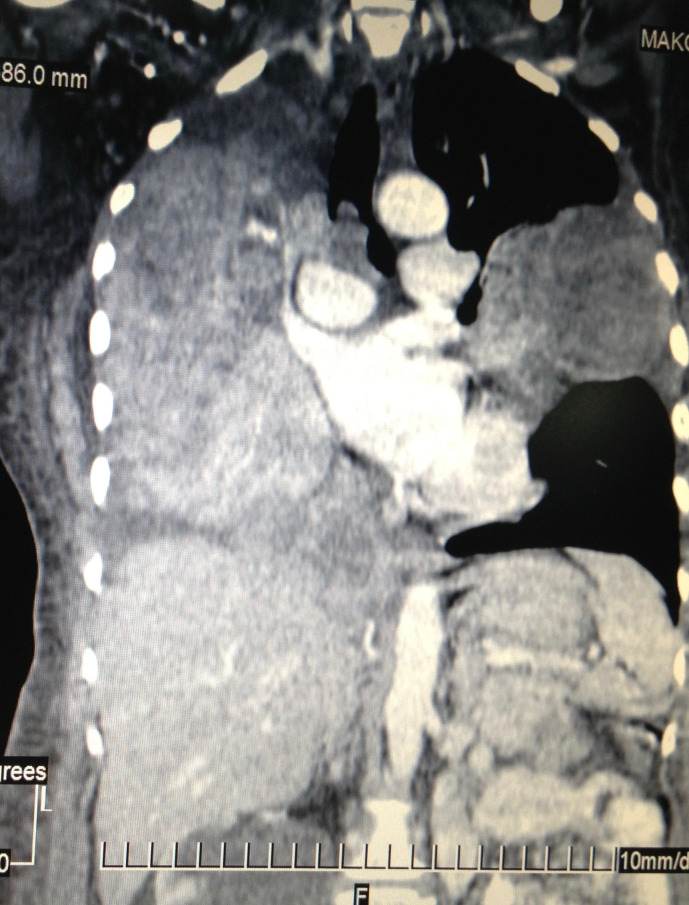
Coronal computed tomography scan showing bilateral heterogeneously enhancing mass lesions.

Mrs AM is a 53-year-old woman from the Democratic Republic of Congo who presented with a 3-month history of generalised body pain. On examination, she had many subcutaneous soft tissue masses. Her chest radiograph and CT chest showed a large rounded mass in the right hemithorax as well as a rounded lesion in the left hemithorax. An ultrasound guided biopsy established a diagnosis of a desmoplastic small round-cell high-grade sarcoma (Figs 5 and 6).

## Discussion

### Malignant mesothelioma

MMs are aggressive neoplasms arising from mesothelial surfaces of the pleura, peritoneal surfaces and tunica vaginalis. Eighty percent will arise from pleural surfaces where 70% of patients will report exposure to asbestos. Other risk factors include radiation, carbon nanotube exposure and genetic factors such as mutations in BRCA1 associated protein 1(BAP1). ^[4–6]^ The common presentation includes marked chest wall pain and pleural effusions often with associated pleural thickening and volume loss. The patient presented uncharacteristically, with pulmonary mass lesions with no identifiable risk factors. The mortality is unfortunately still unacceptably high even with chemotherapy, radiation and surgery

### Adenoid cystic carcinoma

Primary adenoid cystic lung cancers are rare salivary gland neoplasms making up 0.04 - 0.2% of all lung cancers.^[7]^ They are considered slowgrowing tumours, usually arising from the proximal tracheobronchial tree. The solid histological pattern has been associated with a more aggressive clinical course and early distant metastases, in contrast to the cribriform type which shows a more benign behaviour.^[8]^ The mainstay of therapy is surgery. Our patient probably had post obstructive pneumonia and an empyema as a result of the tumour in the left upper lobe.

### Desmoplastic small round cell tumour

Desmoplastic small round cell tumours (DSRCTs) are mesenchymal tumours arising from cells with multi-lineage potential. First described in 1989 by Gerald and Rosai, these tumours have distinct molecular and immunohistochemical characteristics. The molecular hallmark of DSRCT is the Erwing sarcoma and Wilms tumour gene (EWS-WT1) fusion protein.^[9]^ They are characterised histologically by nests of small tumour cells surrounded by cellular and vascular collagenous stroma.^[10,11]^ Mostly arising in the abdominal and pelvic cavity, these tumours can also originate in other sites, such as the lung and pleura.^[12]^ DSRCTs are rare and aggressive malignancies commonly affecting young males with only a few hundred cases reported in the literature. The prognosis is poor and therapy is still not well defined.

## Conclusion

Unfortunately, all 3 patients presented with advanced disease and were referred for oncological assessment and palliative care. This series serves as a reminder of the wide spectrum of presentations of thoracic neoplasms.
